# Supplementary material for: Does ecology shape geographical parthenogenesis? Evidence from the facultatively parthenogenetic stick insect Megacrania batesii
Source: Ecol Evol. 2024 Aug 14;14(8):e70145. doi: 10.1002/ece3.70145 (PMC11322659; doi:10.1002/ece3.70145)
Supplement: Supplementary file 1 — Appendix S1. [file ECE3-14-e70145-s001.docx]

**Appendix:**

Figure A1: Boxplots showing the effect of host plant morphological traits on *M. batesii herbivory*. Traits include a) height of the plant (m), b) leaf thickness (mm), c) leaf toughness (kg/cm²), d) spike length(mm), e) spike angle (mm distance between end of spike and edge of leaf), f) distance between spikes (mm), and g) slenderness ratio (leaf length/leaf width). The lower and upper hinges of the box correspond to the first and third quartiles (25^th^ and 75^th^ percentiles). The median line is shown. The whiskers extend from the hinges to the largest and smallest value within 1.5 times the inter-quartile range. Outlying points are plotted individually. Colour indicates whether *M. batesii* chew-marks were present or absent.

Table A1: Summary of PC loadings for each variable and total proportion of variance explained by each PC axis for A) the morphological variation between plants consumed and ignored by *M. batesii*, B) the chemical variation between plants consumed and ignored by *M. batesii*, C) the morphological variation between plants hosting mixed-sex vs all-female *M. batesii* populations, and D) the chemical variation between plants hosting mixed-sex vs all-female *M. batesii* populations.

| **PC analysis** | **Trait** | **PC1** | **PC2** | **PC3** | **PC4** | **PC5** |
| --- | --- | --- | --- | --- | --- | --- |
| A) Morphological variation between plants consumed and ignored by *M. batesii* | height | -0.1500 | -0.7257 | 0.1503 | 0.6488 | 0.0675 |
|  | Avg_thick | -0.3996 | -0.3263 | -0.2557 | -0.3025 | -0.7068 |
|  | Avg_tough | -0.3460 | -0.3650 | 0.3081 | -0.6110 | 0.5246 |
|  | Spike angle | -0.4256 | 0.3644 | 0.2418 | 0.2952 | 0.1422 |
|  | Spike length | -0.4832 | 0.2393 | 0.0451 | 0.1335 | -0.0767 |
|  | Dist.between spikes | -0.4587 | 0.2090 | 0.1721 | 0.047684 | -0.1058 |
|  | l.w.ratio | 0.2705 | 0.0037 | 0.8527 | -0.08467 | -0.4283 |
|  | **Total proportion explained** | **0.5109** | **0.1738** | **0.1315** | **0.0869** | **0.0491** |
| B) Chemical variation between plants consumed and ignored by *M. batesii* | Carbon | -0.6088 | 0.3551 | 0.7094 |  | |
|  | Hydrogen | -0.6077 | 0.3661 | -0.7048 |  |  |
|  | Nitrogen | 0.5100 | 0.8601 | 0.00708 |  |  |
|  | **Total proportion explained** | **0.7347** | **0.1923** | **0.0731** |  |  |
| C) Morphological variation between plants hosting mixed-sex vs all-female *M. batesii* populations | height | 0.1775 | -0.6634 | 0.4373 | 0.5592 | -0.1205 |
|  | Avg_thick | 0.3997 | -0.2966 | -0.2984 | -0.3743 | -0.6564 |
|  | Avg_tough | 0.3601 | -0.2865 | 0.3629 | -0.5967 | 0.5222 |
|  | Spike angle | 0.3949 | 0.4341 | 0.2494 | 0.3012 | -0.0367 |
|  | Spike length | 0.4797 | 0.2103 | -0.0055 | 0.0431 | -0.2037 |
|  | Dist between spikes | 0.4441 | 0.3170 | 0.0855 | 0.0695 | 0.1423 |
|  | l.w.ratio | -0.3095 | 0.2380 | 0.7201 | -0.3062 | -0.4678 |
|  | **Total proportion explained** | **0.5175** | **0.1669** | **0.1250** | **0.0903** | **0.0459** |
| D) Chemical variation between plants hosting mixed-sex vs all-female *M. batesii* populations | Carbon | 0.6298 | -0.2475 | -0.7363 |  | |
|  | Hydrogen | 0.6052 | -0.4378 | 0.6649 |  |  |
|  | Nitrogen | -0.4869 | -0.8644 | -0.1259 |  |  |
|  | **Total proportion explained** | **0.6185** | **0.2471** | **0.1344** |  |  |

Table A2: Statistical results for permutational regression tests (A,B,D,E) and linear regression model (C) for A) the morphological variation between plants consumed and ignored by *M. batesii*, B) the chemical variation between plants consumed and ignored by *M. batesii*, C) The C/N ratio variation between plants consumed and ignored by *M. batesii*, D) the morphological variation between plants hosting mixed-sex vs all-female *M. batesii* populations, and E) the chemical variation between plants hosting mixed-sex vs all-female *M. batesii* populations. Df is degrees of freedom, SumofSqs is the sum of squares, R2 is the R-squared value, F is the F-value, and Pr is the p-value associated with the F-value. Significant p-values are colored red.

|  |  | **Df** | **SumofSqs** | **R2** | **F** | **Pr** |
| --- | --- | --- | --- | --- | --- | --- |
| A) Morphological variation between plants consumed and ignored by *M. batesii* | *M. batesii*  chewmarks | 1 | 13.56 | 0.022 | 2.642 | 0.044 |
|  | Genus | 1 | 150.58 | 0.244 | 29.347 | 0.001 |
|  | *M. batesii* chewmarks:Genus | 1 | 15.72 | 0.026 | 3.064 | 0.034 |
| B) Chemical variation between plants consumed and ignored by *M. batesii* | *M. batesii* chewmarks | 1 | 42.211 | 0.335 | 23.874 | 0.001 |
|  | Genus | 1 | 9.181 | 0.073 | 5.192 | 0.014 |
|  | *M. batesii* chewmarks:Genus | 1 | 5.653 | 0.045 | 3.197 | 0.066 |
| C) C/N ratio variation between plants consumed and ignored by *M. batesii* | *M. batesii* Chewmarks | 1 | 12.361 | 2.886 | 4.283 | 0.0001 |
|  | Genus | 1 | 16.477 | 7.025 | 2.346 | 0.0242 |
|  | *M. batesii* chewmarks:Genus | 1 | -10.443 | 7.375 | -1.416 | 0.1647 |
| D) Morphological variation between plants hosting mixed-sex vs all-female *M. batesii* populations | *M. batesii* Population type | 1 | 18.31 | 0.043 | 3.5213 | 0.015 |
|  | Genus | 1 | 101.28 | 0.237 | 19.4819 | 0.001 |
|  | *M. batesii* Population type:Genus | 1 | 5.90 | 0.014 | 1.1347 | 0.313 |
| E) Chemical variation between plants hosting mixed-sex vs all-female *M. batesii* populations | *M. batesii* Population type | 1 | 3.226 | 0.03469 | 1.108 | 0.317 |
|  | Genus | 1 | 6.640 | 0.07140 | 2.281 | 0.082 |
|  | *M. batesii* Population type:Genus | 1 | 1.609 | 0.01730 | 0.553 | 0.651 |

Figure A2: PCA results for host plant morphological traits in a) *Pandanus spp.* and b) *Benstonea spp.* Each point represents an individual host plant sample and color indicates whether *M. batesii* chew-marks were present or absent.

Table A3: Coefficients from permutational regression statistical models for the morphological variation between plants consumed and ignored by *M. batesii* in *A) Pandanus spp.* and B) *Benstonea spp.* Columns represent the effect of height of the plant (m), leaf thickness (mm), leaf toughness (kg/cm²), spike angle (mm distance between end of spike and edge of leaf), spike length(mm), distance between spikes (mm), and leaf slenderness ratio (leaf length/leaf width).

|  | Height | Leaf thickness | Leaf toughness | Spike angle | Spike length | Distance between spikes | Slenderness ratio |
| --- | --- | --- | --- | --- | --- | --- | --- |
| A) *Pandanus spp. M. batesii* chewmarks | -0.1885 | -0.0157 | -0.2267 | 0.3224 | 0.2357 | 0.1307 | 0.1243 |
| B) *Benstonea spp. M. batesii* chewmarks | 0.0968 | 0.1416 | -0.1213 | -0.3121 | -0.3434 | -0.2972 | 0.2237 |

Table A4: Statistical results for linear mixed-effect models of the effect of population type (Mixed-sex versus all-female) on density of host plants within a 10x10sq m quadrat, with site of the sample as a random effect. For the fixed effects, Estimate is the estimated coefficients, Std.error is the standard errors of the estimated coefficients, df is degrees of freedom, t-value is the t-value associated with the coefficient estimate and Pr is the p-value associated with the t-value. For the random effects, Variance is the variance estimate, and Std.dev is the standard deviation estimates.

| **Fixed effects** |  |  |  |  |  |
| --- | --- | --- | --- | --- | --- |
|  | Estimate | Std.error | df | t-value | Pr |
| M. batesii Population type | 0.056 | 0.124 | 13.49 | 0.447 | 0.662 |
| **Random effects** |  |  |  |  |  |
|  | Variance | Std.dev |  |  |  |
| Site | 0.230 | 0.480 |  |  |  |
| Residual | 0.019 | 0.138 |  |  |  |

Figure A3: Boxplots showing the relationship between *M. batesii* population type and various host plant morphological traits, including: a) height of the plant (m), b) leaf thickness (mm), c) leaf toughness (kg/cm²), d) spike length(mm), e) spike angle (mm distance between end of spike and edge of leaf), f) distance between spikes (mm), and g) slenderness ratio (leaf length/leaf width). The lower and upper hinges of the box correspond to the first and third quartiles (25^th^ and 75^th^ percentiles). The median line is shown. The whiskers extend from the hinges to the largest and smallest value within 1.5 times the inter-quartile range. Outlying points are plotted individually. Color indicates whether that plant was within an all-female or mixed-sex *M. batesii* population.

Table A5: Multivariate homogeneity of groups dispersions test results, testing the differences in group dispersions between habitats hosting all-female *M. batesii* populations and mixed-sex *M. batesii* populations for a) morphological phenotypic traits and b) chemical phenotypic traits. Df is degrees of freedom, SumofSqs is the sum of squares, Mean sq is the squared mean, F is the F-value, and Pr is the p-value associated with the F-value.

|  |  | Df | SumofSqs | Mean sq | F | Pr |
| --- | --- | --- | --- | --- | --- | --- |
| A) | Groups | 1 | 1.689 | 1.689 | 1.482 | 0.243 |
| Morphology | Residuals | 60 | 68.373 | 1.139 |  |  |
| B) | Groups | 1 | 1.812 | 1.812 | 1.8322 | 0.215 |
| Chemistry | Residuals | 30 | 29.663 | 0.989 |  |  |

Table A6: Statistical results for linear mixed-effect models of the effect of population type (Mixed-sex versus all-female) on group dispersions, with site of the sample as a random effect. For the fixed effects, Estimate is the estimated coefficients, Std.error is the standard errors of the estimated coefficients, df is degrees of freedom, t-value is the t-value associated with the coefficient estimate and Pr is the p-value associated with the t-value. For the random effects, Variance is the variance estimate, and Std.dev is the standard deviation estimates.

| A) | **Fixed effects** |  |  |  |  |  |
| --- | --- | --- | --- | --- | --- | --- |
| Morphological |  | Estimate | Std.error | df | t-value | Pr |
|  | *M. batesii* Population type | -0.1545 | 0.300 | 11.2 | -0.515 | 0.617 |
|  | **Random effects** |  |  |  |  |  |
|  |  | Variance | Std.dev |  |  |  |
|  | Site | 0.200 | 0.447 |  |  |  |
|  | Residual | 0.352 | 0.593 |  |  |  |
| B) | **Fixed effects** |  |  |  |  |  |
| Chemical |  | Estimate | Std.error | df | t-value | Pr |
|  | *M. batesii* Population type | 0.158 | 0.205 | 6.11 | 0.771 | 0.469 |
|  | **Random effects** |  |  |  |  |  |
|  |  | Variance | Std.dev |  |  |  |
|  | Site | 0.041 | 0.204 |  |  |  |
|  | Residual | 0.153 | 0.392 |  |  |  |
